# Gating failure can result in underestimation of cardiac function in myocardial perfusion scintigraphy

**DOI:** 10.1007/s12350-020-02430-8

**Published:** 2020-11-11

**Authors:** Alberto Villagran Asiares, Igor Yakushev, Stephan G. Nekolla

**Affiliations:** 1 Nuklearmedizinische Klinik und Poliklinik Klinikum rechts der Isar der Technischen Universität München, Munich, Germany; 2 Deutsches Zentrum für Herz-Kreislauf-Forschung e.V. Partner site Munich Heart Alliance, Munich, Germany

## Abstract

Here, we present a case with a pacemaker due to an atrioventricular (AV) block 2 Mobitz type, in whom a gating failure resulted in a relevant underestimation of cardiac function in myocardial perfusion scintigraphy. A set of quality control steps for gating errors is proposed.

## Introduction

Myocardial perfusion scintigraphy (MPI) is an established tool in the diagnosis and prognosis of ischemic disease. Apart from perfusion, ECG-gated MPI provides information on cardiac function. Importantly, regional and global systolic left ventricular (LV) function is a powerful prognostic factor on its own.^1^,^2^ In addition, ECG-gated acquisitions are helpful in estimation attenuation artifacts. In ECG-gated MPI, detected events are prospectively synchronized with the electrocardiogram (ECG) to sort the events into a series of contractile phase bins. Specifically, R-waves as detected in the ECG are used to split RR intervals in typically 8 or 16 phases.^3^ A failure in this step can result in a significant underestimation of cardiac function as demonstrated here in a patient with a pacemaker. We propose a set of quality control steps covering technical and imaging aspects.

## Patient Condition

A female patient (77 years old, 165 cm, 68 kg) with a high cardiovascular risk profile and progressive dyspnea was referred to our department for evaluation of suspected ischemic heart disease. She had been diagnosed with cerebral and peripheral vascular disease, diabetes mellitus type II, arterial hypertension, hypercholesterolemia, and nicotine consumption. A two chamber pacemaker due to AV-Block 2 type Mobitz was implanted 25 months ago. A recent echocardiogram report described normal regional and global systolic LV function.

## Protocol

Pharmacological stress was performed with a 47.6 mg adenosine infusion over 5 minutes (0.14 mg⋅kg⋅min) while monitoring the patient with a 12-lead ECG (diagnostic ECG). Initial heart rate was 80 bpm, blood pressure 110/70 mmHg, which increased in 2.5 minutes to 120 bpm when 180 MBq Tc-99m-MIBI Sestamibi were injected. After 107 minutes, the patient underwent the first ECG-gated SPECT acquisition for 15 minutes, 18 minutes later, 534 MBq Tc-99m-MIBI Sestamibi were injected in rest followed with a delay of 15 minutes for the 5 minutes SPECT scan. Both scans were acquired on a D-SPECT camera (Spectrum Dynamics, Caesarea, Israel) in identical upright positions. The MPI acquisition was ECG gated using a 3-lead cardiac triggering monitor (CTM, IVY BIOMEDICAL, Branford, Connecticut, USA). This monitor detects the R-waves in the ECG and sends them as binary pulses to the D-SPECT system for cardiac cycle-counts synchronization. The cardiac cycle was divided into 8 gates.

QGS/QPS package (Cedars-Sinai Medical Center, Los Angeles, CA) was used to MPI visualization and gated MPI analysis: automatic measurement of LV end systolic and end diastolic volumes (ESD and EDV, respectively), and corresponding ejection fraction (EF), and wall motion abnormalities assessment. The analysis was performed using the 17-myocardial AHA segments^4^ in a polar map distribution.

## Results and Discussion

**Figure 1:**
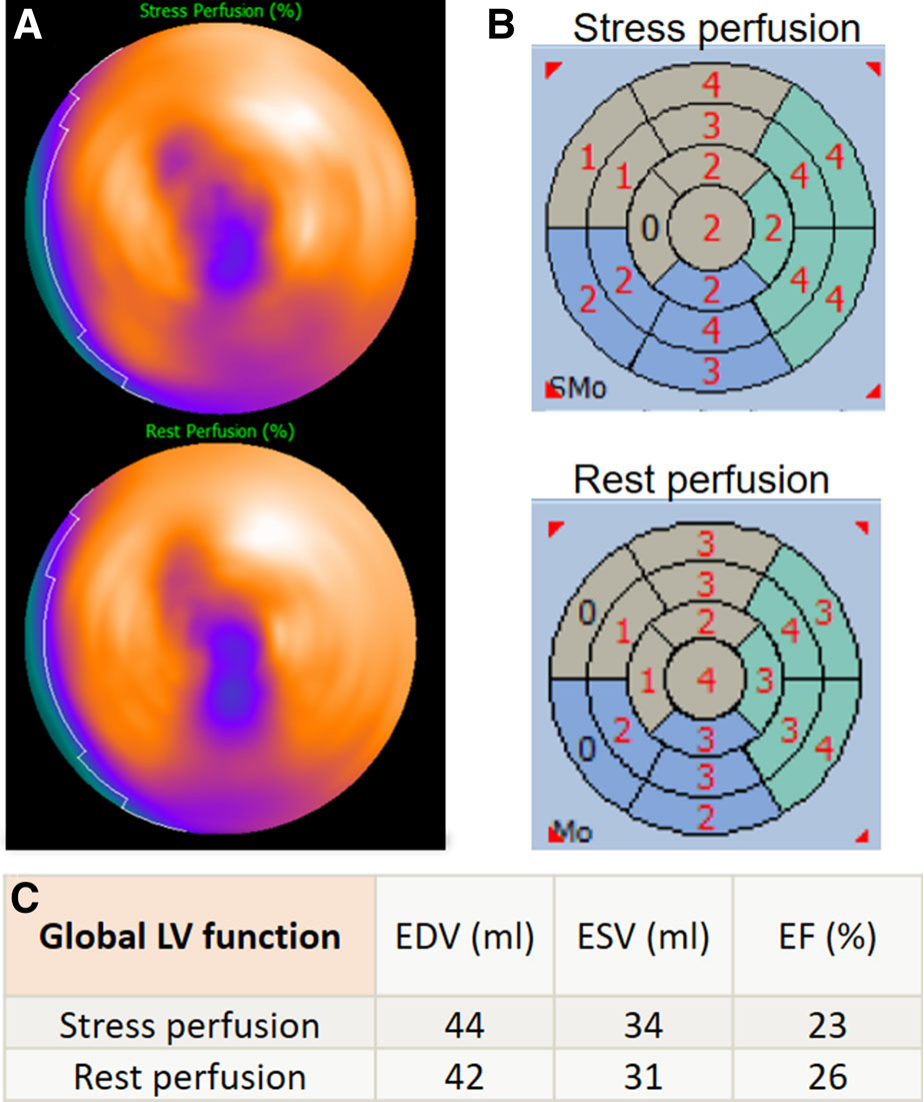
**A** Myocardial perfusion polar maps for stress (top) and rest (bottom) scans. **B** Wall motion abnormalities by gated MPI. **C** Global LV function assessment by end diastolic and end systolic volumes (EDV and ESV) and respective ejection fraction (EF). These results suggest scar in apex region, and severe LV dysfunction with remarked wall motion abnormalities at stress and rest

MPI at stress and rest showed a reduced uptake in the area of apex, indicating either a scar or a breast attenuation artifact. The assessment of LV global and regional function showed a severe dysfunction at stress and rest (ejection fraction below 30%) with a severe global hypokinesia (Figure 1).

**Figure 2:**
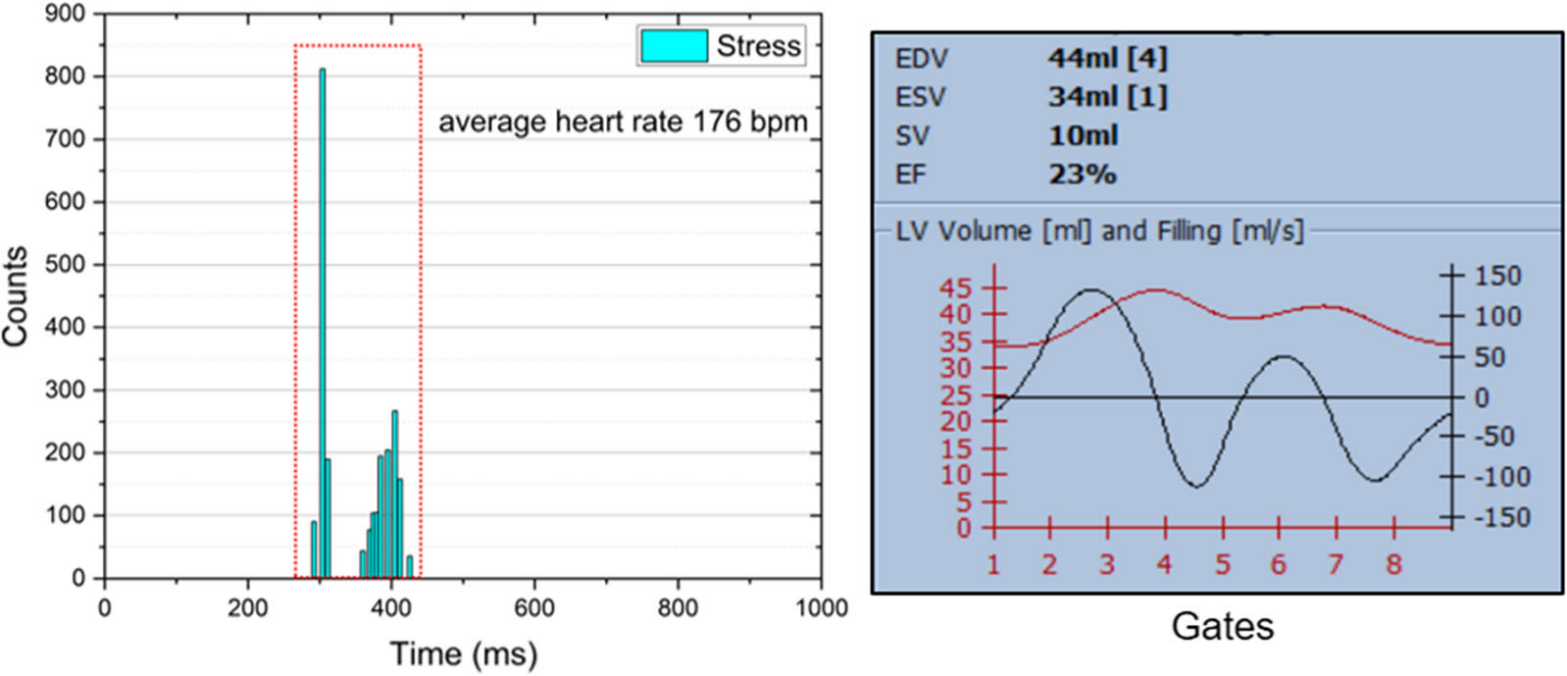
Left panel. Histogram of the cardiac cycles detected for the post-stress acquisition. The two peaks have the same number of counts (1086 counts each). Right panel. LV volume (red) measured in each cardiac phase (gate) together with end diastolic, end systolic and stroke volumes and the corresponding ejection fraction values. The two peaks distribution and mean values of the cardiac cycle do not correspond to the pattern seen in the diagnostic ECG and previous studies. This reconstruction led to low EF (the rest analysis is not shown but features basically identical behavior)

As these results were inconsistent with the clinical impression and the echocardiogram, concerns were raised by a physician. Already at first glance, the ECG-gated SPECT-based LV volumes curves (Figure 2) show two myocardial cycles. In addition, the heart rate reported by the SPECT system differed from the diagnostic ECG. Further investigation revealed unusual beat histograms. Figure 2 shows the beat histograms used for stress and rest ECG-gated SPECT. The histograms present two peaks with an equal number of counts each, centered at approx. 300 ms (HR: 200 bpm) and 400 ms (HR: 150 bpm) in both scans. These values differed from the heart rates manually measured from the 12-lead stress ECG signal: 80 bpm at rest state, and maximum heart rate 120 bpm at stress. It is worth mentioning that both ECG systems (12-lead and 3-lead) had problems with measuring the correct heart rate: even from the 12-lead ECG, the automatically computed value differed from the manually measured value, reporting a heart rate twice higher.

**Figure 3:**
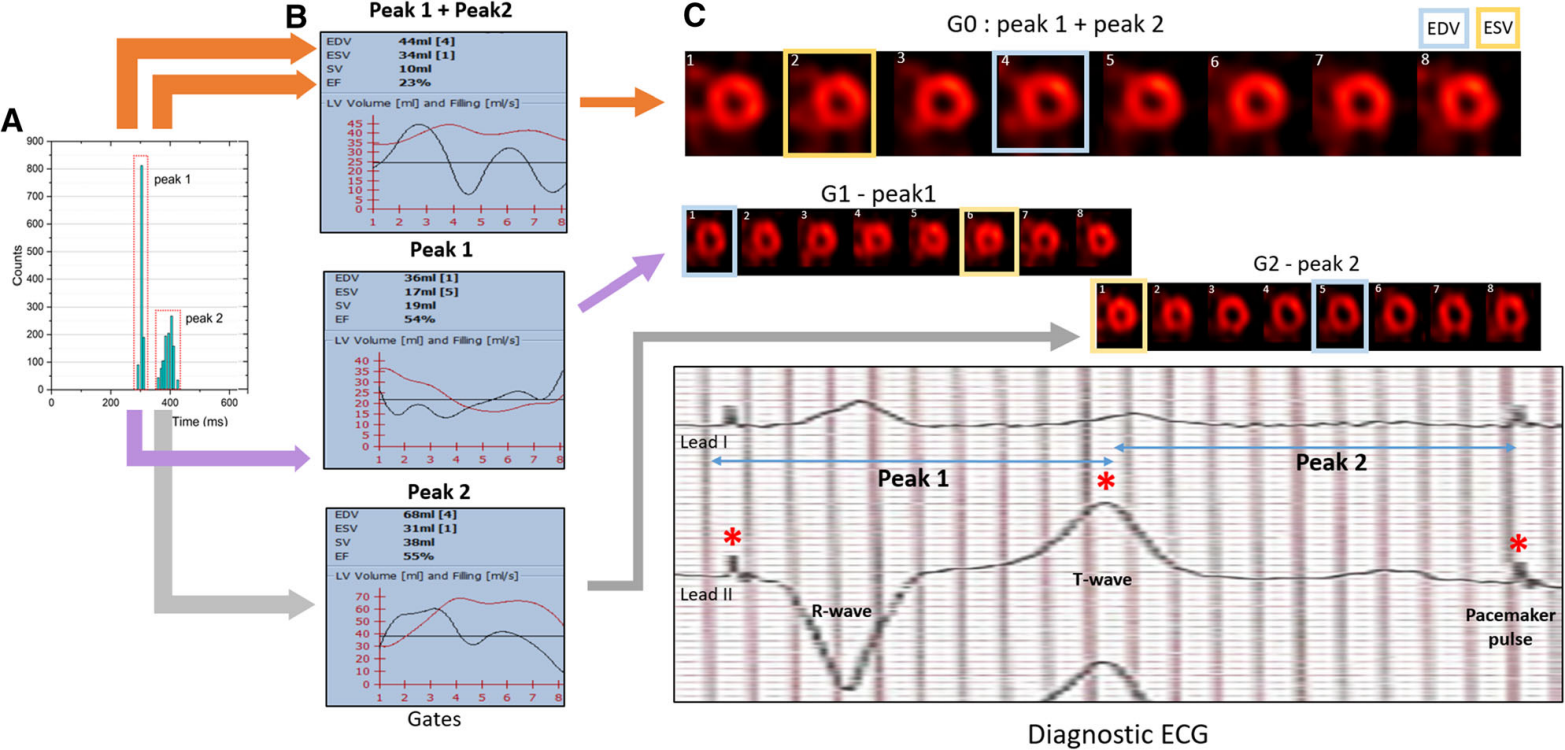
**A** Histogram of the “pseudo beats” detected by the camera’s cardiac trigger device for gated MPI. The beat acceptance window (red) was centered on each peak to reconstruct then the corresponding gated MPI (G1 and G2). **B** LV volume curves at post-stress (red) obtained from the images reconstructed using both peak (top), only peak 1 (mid), and only peak 2 (bottom). **C** Top. Gated MPI images obtained with peak1 plus peak 2 (G0), only with peak 1 (G1), and with peak 2 (G2). Bottom. Pre-adenosine diagnostic ECG (lead I and II), showing the signals pacemaker pulse and T-wave (*) that would be detected as R-wave (“pseudo R-waves”) by the trigger device. G1 + G2 follow the contractile pattern of a typical cardiac cycle compatible with the clinical history of this patient

This finding suggests that the delineation of the cardiac cycle was incorrect: the CTM would have wrongly triggered after the detection of intracycle waves, instead of the R-waves. Particularly, the ECG of the patient recorded an elevated T-wave that was wrongly detected for an R-wave (Figure 3). Then, since a beat acceptance window including all the waves (peaks) detected was initially used during the gating processing, the contractile function registered by MPI disagreed with the clinical history.

Interestingly, the initial attempt to select only one of these peaks still did not solve the problem, as discussed in the following: due to the intracycle “pseudo R-waves” detection, no matter what beat rejection window used, the true heart rate could not be set in the beat histogram for ECG-gated image reconstruction. This disabled a reliable global and regional LV function assessment.

Nevertheless, in order to estimate LV volumes and corresponding EF, we manually integrated the results from the ECG-gated series reconstructed from both peaks. As the triggering split the cardiac cycle virtually into two overlapping parts, a combined LV volume curve was constructed from the two ECG-gated scans (Figure 3). Consequently, we selected the largest diastolic and the smallest systolic volume. Thus, the values for EDV, ESV, and EF at stress were 68 mL, 17 mL, and 75 %, respectively, while at rest 66 mL, 19 mL, and 72%. Thus, this estimation suggests that the global LV function of this patient is within the normal range, contrary to what was seen initially.

Since the incorrect identification of the “true” R-wave led potentially to a misdiagnosis it is important to highlight checkpoints and solutions to avoid this error since no “correct” beat histogram can be selected.

The following quality control should be done at the step of data acquisition. First the heart rate on the ECG system of the imaging device should be compared with that on the diagnostic ECG device, ensuring similar values. Second, quality of the trigger signal should be checked with a special attention in the case of an abnormal ECG (e.g., S or T-wave alterations), and settings should be adjusted if needed (e.g., lead change or specific features of the ECG such as a p-lock function activation for this particular device^5^). Recording the signal is highly recommended. Lastly, at the step of data processing, heart rate values and distribution, as well as a correct position of the beat acceptance window in the histogram of the gating quality control module should be compared with the heart rate from the 12-lead ECG.

## Conclusion

We describe the clinically relevant impact of synchronization failure on assessment of cardiac contractile function in myocardial perfusion scintigraphy. Such gating errors can result in underestimation of regional and global LV function, especially in patients with pacemakers. A quality control of the gating process during myocardial scintigraphy is essential.
